# Retrospective Analysis of Cement Extravasation Rates in Vertebroplasty, Kyphoplasty, and Bone Tumor Radiofrequency Ablation

**DOI:** 10.3390/jcm14092908

**Published:** 2025-04-23

**Authors:** Soun Sheen, Prit Hasan, Xiaowen Sun, Jian Wang, Claudio Tatsui, Kent Nouri, Saba Javed

**Affiliations:** 1 Department of Pain Medicine, The University of Texas MD Anderson Cancer Center, Houston, TX 01605, USA; 2 Department of Orthopedics and Physical Rehabilitation, The University of Massachusetts Chan Medical School, Worcester, MA 01610, USA; 3 Department of Biostatistics, The University of Texas MD Anderson Cancer Center, Houston, TX 77006, USA; 4 Department of Neurosurgery, The University of Texas MD Anderson Cancer Center, Houston, TX 77006, USA

**Keywords:** vertebral compression fractures, vertebral augmentation, vertebroplasty, kyphoplasty, bone-tumor radiofrequency ablation, cancer pain

## Abstract

**Background:** Percutaneous vertebral augmentation techniques, including vertebroplasty, kyphoplasty, and bone tumor radiofrequency ablation (BT-RFA), are commonly used to treat painful vertebral compression fractures (VCFs). While generally safe and effective, they carry risks, including cement extravasation, which can lead to pulmonary embolism or spinal cord compression. This study aims to compare the rate of cement extravasation across different vertebral augmentation techniques and identify potential risk factors. **Methods:** A retrospective cohort study was conducted at a comprehensive cancer center on 1002 procedure encounters in 888 patients who underwent vertebral augmentation for painful VCFs. Data were collected on patient demographics, fracture pathology, procedure type, imaging guidance, and pain scores. Intraoperative and postoperative imaging were manually reviewed to assess cement extravasation. Statistical analyses were performed using pairwise comparisons with Tukey’s Honest Significant Difference adjustment to compare cement extravasation rates across the procedure groups and generalized linear mixed models to assess the association between the cement extravasation with other variables. **Results**: Cement extravasation occurred in 573 (57.2%) encounters. Kyphoplasty had the lowest rate of cement extravasation (46.2%) with significantly lower odds compared to vertebroplasty (OR: 0.42, 95% CI: 0.30–0.58; *p* < 0.0001) and BT-RFA (OR: 0.57, 95% CI: 0.42–0.77; *p* = 0.0009). Pathologic fractures and multilevel augmentations were linked to a 64% (*p* = 0.001) and 63% (*p* = 0.0003) increased odds of cement extravasation, respectively. Male sex and older age were protective factors. **Conclusions:** Cement extravasation is a common but largely asymptomatic complication of percutaneous vertebral augmentation. It is crucial to consider patient-specific risk factors when selecting an augmentation technique to optimize outcomes. Kyphoplasty may be the optimal choice for patients at increased risk of cement extravasation.

## 1. Introduction

Vertebral compression fractures (VCFs), whether pathologic or osteoporotic, are common, affecting approximately 1.5 million patients annually in the United States [1]. These fractures can be highly debilitating, leading to acute and chronic pain, reduced physical function, decreased quality of life, and even increased mortality [2,3,4]. For patients with painful VCFs poorly responsive to conservative care, percutaneous vertebral augmentation has become a widely used, minimally invasive treatment option to not only alleviate pain and improve function but also stabilize vertebral fractures and restore vertebral height [5,6,7].

There are several vertebral augmentation techniques available, each with unique procedure elements. Vertebroplasty involves a direct placement of bone cement, typically polymethylmethacrylate, into the vertebral body. Kyphoplasty, by contrast, creates a cavity within the vertebral body using a balloon or other expandable device, such as jacks (SpineJack), prior to injecting the cement. Bone tumor radiofrequency ablation (BT-RFA) utilizes thermal ablation of spinal tumors and is typically performed in conjunction with cement augmentation. These procedures have been established to be effective and safe [5,6,7,8,9,10].

Despite its established efficacy and safety, vertebral augmentation is associated with complications such as cement extravasation, adjacent vertebral fractures, or neurologic injury [2,3]. Cement extravasation is among the most common complications, with reported rates ranging from less than 10% to as high as 90% [2,6]. This wide variability highlights inconsistencies in the current literature, possibly due to inconsistent detection methods and criteria. Furthermore, as the vast majority of cement extravasations are clinically asymptomatic, they are often considered inherent and inevitable in vertebral augmentation [11]. However, cement extravasation can lead to potentially fatal complications including pulmonary embolism or spinal cord compression; therefore, it is crucial to identify risk factors to help mitigate these risks. 

There is some evidence suggesting that newer augmentation techniques, such as kyphoplasty or BT-RFA, may reduce the risk of cement extravasation by allowing low-pressure cement injection by cavity formation [10,12]. However, due to inconsistencies in the literature, a definitive comparison of cement extravasation rates across augmentation techniques is lacking. In light of the increasing utilization of vertebral augmentation and the expanding variety of augmentation options, a comprehensive assessment of extravasation risks is timely and necessary. 

Our study aims to address this gap by comparing extravasation rates among different vertebral augmentation techniques and identifying potential risk factors associated with cement extravasation to offer further insights into procedural approaches that could enhance patient safety and treatment outcomes in vertebral augmentation.

## 2. Methods

### 2.1. Study Design

This is a single-center, descriptive retrospective cohort study conducted at an academic, tertiary-care comprehensive cancer center. This study was conducted in accordance with the Declaration of Helsinki, and the protocol was reviewed and approved by the institutional review board (#2024-0530), and the study data were searched using the institutional Epic medical record system. Informed consent was waived as the study involved no more than minimal risks to subjects as there was no change in patient care.

**Figure 1:**
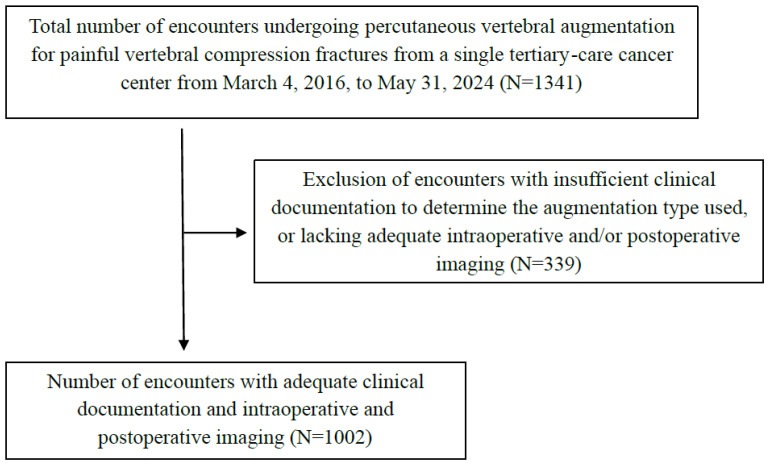
Flow diagram of the patient selection process.

For each procedure encounter, intraoperative and postoperative imaging studies were manually reviewed by two experienced clinical investigators (S.S. and S.J.), along with radiologist reports, to assess cement extravasation. Postoperative imaging studies were limited to CT cervical, thoracic or lumbar spine, CT chest, or CT abdomen and pelvis within 6 months of the procedure. Encounters without adequate intraoperative or postoperative imaging studies to accurately determine the presence of cement extravasation were excluded (Figure 1).

### 2.2. Study Participants

Eligible patients were at least 18 years of age who underwent percutaneous vertebral augmentation procedures for painful vertebral compression fractures between 4 March 2016, to 31 May 2024. In order to minimize selection bias, all procedures performed by a range of providers, including pain specialists, interventional radiologists, and neurosurgeons, were included. Encounters without adequate intraoperative or postoperative imaging studies to accurately determine the presence of cement extravasation were excluded (Figure 1). If a patient underwent combined treatment cases (i.e., multiple types of augmentations performed within the same encounter), only the initially planned augmentation technique and the vertebral levels associated with that specific procedure were included. Cases were excluded if the clinical documentation did not provide sufficient detail to determine the specific type of vertebral augmentation performed at each level.

### 2.3. Outcomes

The primary outcome was the incidence of cement extravasation. The secondary outcome measures included the clinical significance of extravasation, including but not limited to neurologic or vascular complications, (followed up for 6 months) and pain scores rated on an 11-point numeric rating scale (NRS) within 14 days of the procedure. The following demographic and clinical variables were collected from the electronic medical record: age, sex, race, body mass index (BMI), primary cancer diagnosis, types of vertebral augmentation performed (vertebroplasty, kyphoplasty with balloon or SpineJack, or BT-RFA with vertebroplasty or kyphoplasty), type of imaging guidance used (CT, fluoroscopy, or CT combined with fluoroscopy), number of levels treated (single or multilevel), presence of vertebral malignant lesions at the levels performed (yes or no), height loss greater than 50% at the level of cement extravasation (yes or no), and location of cement extravasation (intervertebral disc space, paravertebral soft tissues or veins, posterior (spinal canal or neuroforamen), or multidirectional including more than one of the aforementioned directions). If a patient underwent combined treatment cases (i.e., multiple types of augmentations performed within the same encounter), only the initially planned augmentation technique and the vertebral levels associated with that specific procedure were included. Cases lacking adequate documentation of the exact type of augmentation used were excluded.

### 2.4. Analysis

Descriptive statistics were used to summarize the clinical and demographic characteristics at both the patient and procedure levels. Continuous variables (e.g., age, BMI) were summarized using means and standard deviations (SD). Categorical variables (e.g., sex) were summarized using frequencies and proportions. At the patient level, the average age and BMI across different procedures for each patient were used to calculate summary statistics. To account for the correlation of repeated procedures within patients (i.e., 1002 procedures in 888 patients), a generalized linear mixed model was used to compare variables between the cement extravasation and no cement extravasation groups. Specifically, cement extravasation was the outcome, variable of interest was included as a fixed effect, and patient ID was included as a random effect to account for within-patient correlation. Pairwise comparisons were performed to assess cement extravasation risks across different procedure groups (vertebroplasty, kyphoplasty, and BT-RFA) and imaging guidance groups (CT, fluoroscopy, and CT with fluoroscopy), with Tukey’s Honest Significant Difference adjustment applied for multiple comparisons. A multinomial generalized estimating equations model was used to associate the direction of cement extravasation and the procedure groups. Pre- and post-procedural NRS pain scores were compared using a linear mixed effect model. Multivariable analysis was not performed due to the exploratory nature of the study. A two-sided *p*-value of <0.05 was considered statistically significant. A simulation-based post-hoc sample size justification was also conducted retrospectively. Using 1000 simulated data sets, each with 888 patients and 1002 total procedures (approximately 1.13 procedures per patient), we estimated that the study had approximately 80% power to detect an odds ratio of 0.65 or 1.54 at a two-sided significance level of 0.05. The simulation incorporated an intra-class correlation coefficient of 0.1, reflecting the within-patient variability observed in the dataset. All statistical analyses were conducted using R Statistical Software (v4.2.1; R Core Team 2022). 

## 3. Results

A total of 1341 encounters were identified in 1117 patients who underwent percutaneous vertebral augmentations from 4 March 2016 to 31 May 2024. After excluding 339 encounters (229 patients) due to inadequate information for accurately determining the augmentation used or the presence of cement extravasation, 1002 encounters in 888 patients were included in the analysis. The mean (SD) age was 64.3 (12.4) years, with 434 (48.9%) patients being female (Table 1). Among the 888 patients, 712 (80.2%) had active cancer, 171 (19.3%) had a history of cancer, and 5 (0.5%) had no cancer history. The most prevalent types of cancer among the patients included multiple myeloma (13.7%), breast cancer (12.8%), prostate cancer (12.6%), lung cancer (11.9%), and renal cancer (10.7%) (Table 1). Of the 1002 procedure encounters, 774 (77.4%) involved pathologic fractures due to malignant vertebral lesions, while 226 encounters (22.6%) involved non-pathologic fractures (Table 2).

**Figure 2:**
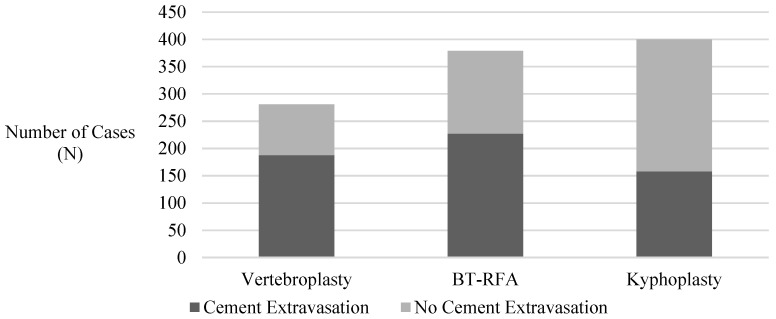
Incidence of cement extravasation observed in each augmentation technique. Abbreviations: BT-RFA, bone tumor radiofrequency ablation.

Among the 1002 procedure encounters, cement extravasation was observed in 573 (57.2%) cases, with multiple vertebral levels involved in 20 (3.5%) cases. Approximately one-third of the extravasation cases occurred in vertebral bodies with greater than 50% height loss. Cement extravasation was observed in 188 of 281 vertebroplasty procedures (66.9%), 227 of 379 BT-RFA procedures (59.9%), including 305 combined with kyphoplasty and 75 combined with vertebroplasty, and 158 of 342 kyphoplasty procedures (46.2%), which included 224 with balloon and 118 with SpineJack (Figure 2). 

Pairwise comparisons among the procedure types demonstrated that kyphoplasty was associated with the lowest odds of cement extravasation (Table 3). Kyphoplasty was associated with approximately 58.1% lower odds of cement extravasation compared to vertebroplasty (odds ratio (OR): 0.42, 95% confidence interval (CI):0.30–0.58; *p* < 0.0001), and 43.0% lower odds compared to BT-RFA (OR: 0.57, 95% CI:0.42–0.77; *p* = 0.0009). No statistically significant relationship was observed between vertebroplasty and BT-RFA. Additionally, there was no significant difference in cement extravasation between BT-RFA with kyphoplasty and with vertebroplasty.

Of the 573 cases of cement extravasation, 465 (81.2%) involved pathologic VCFs due to malignant lesions at the augmentation site. The incidence of cement extravasation was higher in pathologic VCFs (59.9%) compared to non-pathologic VCFs (47.8%). Pathologic VCFs were associated with a 1.64-fold increase in the odds of cement extravasation (OR: 1.64, 95% CI: 1.21–2.21; *p* = 0.001) (Table 2). Additionally, multilevel vertebral augmentations were also linked to a 1.63-fold increase in the odds of cement extravasation (OR: 1.63, 95% CI: 1.26–2.13; *p* = 0.0003). 

In contrast, males exhibited approximately a 34% lower odds of cement extravasation (OR: 0.66, 95% CI: 0.51–0.86; *p* = 0.002). Furthermore, significantly lower odds of cement extravasation were observed in older patients, particularly for those aged 60 and above (Table 2). For each one-year increase in age, a 2% reduction in the odds of cement extravasation was observed (*p* < 0.05). No significant associations were found for BMI, concurrent osteopenia or osteoporosis, or type of imaging guidance (CT, fluoroscopy, or CT combined with fluoroscopy) (Table 2 and Table 4). 

**Figure 3:**
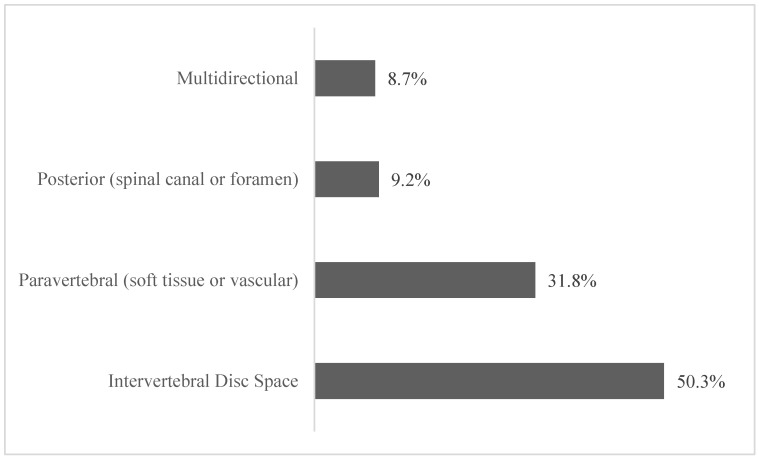
Cement extravasation location.

Intervertebral disc extravasation was the most prevalent (50.3%, *n* = 288), followed by paravertebral extravasation (31.8%, *n* = 182), and posterior extravasation (9.2%, *n* = 53). Fifty cases (8.7%) involved extravasation into more than one space (Figure 3). The type of procedure did not have a statistically significant effect on the location of extravasation (Table 5). 

The vast majority of cement extravasations, including those into vascular structures and the spinal canal and neuroforamen, were asymptomatic. However, one case of extravasation into the inferior vena cava (IVC) resulted in pulmonary emboli, leading to patient death. Another case involving posterior extravasation into the neuroforamen caused severe radicular pain, and the patient was not a candidate for surgical removal due to the extent of cement involvement. In another case of vascular extravasation, the cement embolus lodged in a previously placed IVC filter, but the patient remained asymptomatic. For pain outcomes, the vertebroplasty cohort demonstrated a statistically significant reduction in pain scores from pre- to post-procedure while no significant difference in pain scores was observed overall or within the kyphoplasty and BT-RFA cohorts (Table 6). 

## 4. Discussion

Cement extravasation is a common complication in vertebral augmentation, with reported rates as high as 90%. However, reported rates in the literature are inconsistent, likely due to the minimal clinical significance of most cases and the variability in imaging modalities used to detect extravasation. In our study, cement extravasation was observed in 57.2% across various vertebral augmentation techniques based on intraoperative and postoperative imaging studies. In our study, both intra-operative images as well as postoperative CT scans with higher resolution were reviewed. This detection method allowed for more accurate detection, but it may have also led to a higher rate of cement extravasation than previously reported. Consistent with existing literature, intradiscal and paravertebral extravasations comprised approximately 80% of cement extravasation cases. Notably, only two of the 573 (0.35%) patients with cement extravasation were symptomatic.

Kyphoplasty, when compared to vertebroplasty and BT-RFA, demonstrated decreased odds of cement extravasation. Previous studies have shown that kyphoplasty is associated with a lower risk of extravasation than vertebroplasty, which may be attributed to the unique mechanics of kyphoplasty involving balloon inflation or mechanical jack expansion within the vertebral body [5,13,14,15,16,17,18]. In addition to restoring vertebral height, these techniques create a cavity that not only helps to contain the cement within the vertebral body but also allows for a lower-pressure injection [12,19]. However, despite this advantage, there is conflicting evidence, with some studies reporting higher extravasation rates in vertebroplasty possibly related to higher cement volumes [20,21] or no significant difference between the two groups [6,22]. While the cavity creation in kyphoplasty may prevent extravasation, it has been suggested that balloon or jack inflation could potentially lead to iatrogenic endplate damage and subsequent cement extravasation [23].

BT-RFA has also been considered a viable alternative for pathologic VCFs. By ablating the lesion, BT-RFA not only allows for a lower-pressure cement distribution but also reduces vascular leakage by thrombosing the intravertebral venous plexus [23]. Furthermore, in an animal model study, a dense layer formation at the tumor edge was observed with thermal ablation, creating a biomembrane barrier that may potentially reduce cement extravasation [24]. While Bornemann et al. demonstrated a significantly lower risk of cement extravasation in BT-RFA compared to kyphoplasty [8], this finding was not supported by our study. Although the overall rate of cement extravasation for BT-RFA was lower than vertebroplasty, no statistical significance was observed.

Pathologic VCFs were linked to a 64% increased odds of cement leakage, which aligns with earlier research findings [19,25,26,27]. This may be attributed to greater tumor-related destruction of the vertebral cortex or pedicle. Despite the higher extravasation risk in pathologic VCFs, the overall incidence of symptomatic extravasation or fatal complications remains low [26,28]. Although our study did not demonstrate a reduced risk of cement extravasation with BT-RFA, other studies have demonstrated that the history of prior cancer treatment to the affected vertebra, such as radiation, embolization, or ablation (radiofrequency or cryoablation) correlated with a decreased risk of cement extravasation [26,29]. Especially given the increased comorbidities often associated with malignancy, careful patient selection is essential to reduce the overall risk of cement extravasation in this population.

Male sex and older age, particularly those 60 years or older, were found to be protective against cement extravasation in this study. There is limited evidence on the association between sex and cement extravasation, but our study supports previous findings suggesting that females may have a higher risk of extravasation [30,31]. Prior studies have also produced inconsistent results regarding age and extravasation risk. Shi et al., in a retrospective study of 308 patients with metastatic spinal disease, found that younger patients were at a higher risk [29]. It is speculated that higher bone density in younger patients may require higher pressure injection and cement volumes, potentially leading to increased risk of cement extravasation. On the other hand, older patients, particularly those with more advanced degenerative disc disease, likely have more sclerotic endplates, decreased disc space, and stiffer discs, which may limit the likelihood of intradiscal extravasation.

While vertebroplasty significantly reduced pain, this effect was not observed with kyphoplasty and BT-RFA, potentially adding to the controversy surrounding the effectiveness of vertebral augmentation techniques. However, it is important to note that post-procedure pain scores were recorded from the first encounter within 14 days post-procedure. This limited follow-up period may not fully capture the effects of more extensive techniques like kyphoplasty and BT-RFA (i.e., larger trocars, bilateral approach), which may require additional time for measurable pain relief. Consequently, the short-term post-procedure pain data in our study may underestimate the impact of kyphoplasty and BT-RFA on pain relief, suggesting the need for longer follow-up intervals to assess pain outcomes more accurately.

The majority of cement extravasations are asymptomatic, which may contribute to a perception that extravasation has limited clinical significance. However, cement extravasation can result in serious complications, including potentially fatal outcomes such as pulmonary embolism that caused patient mortality in this study. Thus, continued efforts are crucial to identify risk factors and minimize cement extravasation. Our findings suggest that kyphoplasty may benefit patients at higher risk for extravasation. In patients with pathologic VCFs or multilevel fractures, thorough preoperative assessment is crucial to identify and minimize risks.

To our knowledge, this study is the first to compare cement extravasation risks across multiple vertebral augmentation techniques. Our findings highlight distinct risk factors that may lead to cement extravasation, which can be fatal in some cases. Future research is warranted to clarify the association between cement extravasation risk and patient and fracture characteristics. Furthermore, the development of a more comprehensive and standardized preoperative algorithm is needed to improve risk assessment and procedural planning. Enhanced intraoperative and postoperative monitoring protocols may also be beneficial for patients at higher risk.

This study is not without limitations. Due to the nature of retrospective studies, it is subject to unquantifiable sources of biases that may have influenced the results. The retrospective use of an electronic database could also introduce biases and errors. As the study was completed at a tertiary cancer center, all but five patients had a history of active or prior cancer. Although the study also includes patients with non-pathologic VCFs, we recognize that the study cohort is unique and the generalizability of the study results may be limited. Given the unique characteristics of our study population, future studies evaluating the risks of cement extravasation specifically in osteoporotic fractures would be important to broaden applicability. Furthermore, as a large percentage of VCFs were pathologic, the study results should be interpreted with caution. Lastly, adjustments for multiple comparisons were not conducted for all analyses. Future independent studies are needed to further validate our findings.

## 5. Conclusions

Cement extravasation is a common yet significantly underappreciated complication of vertebral augmentation. Although most cases are asymptomatic, it is important to consider the risk factors preoperatively. Our findings indicate that pathologic VCFs, multilevel augmentation, female sex, and younger age are associated with a higher risk of cement extravasation. Kyphoplasty demonstrated the lowest risk of cement extravasation when compared to vertebroplasty and BT-RFA, suggesting it may be the optimal choice for patients at increased risk. Further prospective studies are needed to identify additional risk factors to refine patient selection and minimize the risk of cement extravasation.

## Figures and Tables

**Table 1:** Baseline characteristics of the overall cohort at the patient level.

Variable	Overall Cohort (*n* = 888)
Age	
Mean (SD), years	64.3 (12.4)
Sex	
Female, *n* (%)	434 (48.9%)
Male	454 (51.1%)
BMI	
Mean (SD), kg/m^2^	27.4 (6.2)
Race	
American Indian or Alaska Native	7 (0.8%)
Asian	39 (4.4%)
Black or African American	82 (9.2%)
Caucasian or White	702 (79.1%)
Native Hawaiian or Other Pacific Islander	2 (0.2%)
Other	51 (5.7%)
Unknown	5 (0.6%)
Primary Cancer Diagnosis	
Bladder Cancer	26 (2.9%)
Breast Cancer	114 (12.8%)
Colorectal Cancer	41 (4.6%)
Esophageal/Gastric Cancer	25 (2.8%)
Gynecologic Cancer	25 (2.8%)
Head and Neck Cancer	23 (2.6%)
Kidney Cancer	95 (10.7%)
Leukemia	18 (2.0%)
Liver/Gallbladder Cancer	26 (2.9%)
Lung Cancer	106 (11.9%)
Lymphoma	24 (2.7%)
Multiple Myeloma	122 (13.7%)
Myelofibrosis/ Myelodysplastic Syndrome	14 (1.6%)
Pancreatic Cancer	26 (2.9%)
Prostate Cancer	112 (12.6%)
Sarcoma	25 (2.8%)
Skin Cancer	39 (4.4%)
Thyroid Cancer	10 (1.1%)
Other	12 (1.4%)
No Cancer History	5 (0.6%)

**Table 2:** Comparison of cement extravasation rates between covariates. Summary statistics were calculated at the procedure level.

	Overall Cohort (*n* = 1002)	Cement Extravasation (*n* = 573)	No Cement Extravasation (*n* = 429)	OR	95% CI	*p*-Value
Age						
Mean (SD), years	64.6 (12.3)	63.4 (12.5)	66.2 (11.9)	0.98	0.97–0.99	**0.0005**
Age groups						
<40 ^a^, *n* (%)	42 (4.2%)	30 (5.2%)	12 (2.8%)			
40–49	69 (6.9%)	45 (7.9%)	24 (5.6%)	0.75	0.33–1.73	0.50
50–59	188 (18.8%)	122 (21.3%)	66 (15.4%)	0.74	0.35–1.54	0.42
60–69	326 (32.5%)	178 (31.1%)	148 (34.5%)	0.48	0.24–0.97	**0.04**
70–79	289 (28.8%)	155 (27.1%)	134 (31.2%)	0.46	0.23–0.94	**0.03**
80 or older	88 (8.8%)	43 (7.5%)	45 (10.5%)	0.28	0.17–0.02	**0.02**
Sex						
Female ^a^, *n* (%)	490 (48.9%)	305 (53.2%)	185 (43.1%)			
Male	512 (51.1%)	268 (46.8%)	244 (56.9%)	0.66	0.51–0.86	**0.002**
BMI						
Mean (SD), kg/m^2^	27.4 (6.2)	27.4 (6.4)	27.4 (5.9)	1.00	0.98–1.02	0.93
Fracture type						
Non-pathologic ^a^, *n* (%)	226 (22.6%)	108 (18.8%)	118 (27.5%)			
Pathologic	776 (77.4%)	465 (81.2%)	311 (72.5%)	1.64	1.21–2.21	**0.001**
Levels						
Single ^a^, *n* (%)	587 (58.6%)	307 (53.6%)	280 (65.3%)			
Multilevel	415 (41.4%)	266 (46.4%)	149 (34.7%)	1.63	1.26–2.13	**0.0003**
Bone Density ^b^						
Normal ^a^, *n* (%)	33 (3.3%)	20 (3.5%)	13 (3.0%)			
Osteopenia	90 (9.0%)	55 (9.6%)	35 (8.2%)	1.02	0.45–2.32	0.96
Osteoporosis	111 (11.1%)	54 (9.4%)	57 (13.3%)	0.62	0.28–1.36	0.23
Unknown	768 (76.6%)	444 (77.5%)	324 (75.5%)	0.89	0.44–1.82	0.75

Abbreviations: CI, confidence interval; DEXA, dual x-ray absorptiometry; OR, odds ratio. ^a^: Reference level used if applicable. ^b^: Bone density determined by DEXA scans. Bolded *p*-values indicate statistical significance.

**Table 3:** Comparison of cement extravasation rates among the augmentation types. Summary statistics were calculated at the procedure level.

	Overall Cohort (*n* = 1002)	Cement Extravasation (*n* = 573)	No cement Extravasation (*n* = 429)	OR	95% CI	*p*-Value ^b^
Kyphoplasty vs. BT-RFA						
BT-RFA ^a^, *n* (%)	379 (37.8%)	227 (39.6%)	152 (35.4%)			
Kyphoplasty	342 (34.1%)	158 (27.6%)	184 (42.9%)	0.57	0.42–0.77	0.0009
Kyphoplasty vs. Vertebroplasty						
Vertebroplasty ^a^, *n* (%)	281 (28.0%)	188 (32.8%)	93 (21.7%)			
Kyphoplasty	342 (34.1%)	158 (27.6%)	184 (42.9%)	0.42	0.30–0.58	<0.0001
Vertebroplasty vs. BT-RFA						
BT-RFA ^a^, *n* (%)	379 (37.8%)	227 (39.6%)	152 (35.4%)			
Vertebroplasty	281 (28.0%)	188 (32.8%)	93 (21.7%)	1.36	0.98–1.89	0.1577

Abbreviations: BT-RFA, bone tumor radiofrequency ablation; CI, confidence interval; OR, odds ratio. ^a^: Reference level. ^b^: *p*-value was adjusted for multiple comparisons.

**Table 4:** Comparison of cement extravasation rates among the imaging guidance types. Summary statistics were calculated at the procedure level.

	Overall Cohort (*n* = 1002)	Cement Extravasation (*n* = 573)	No Cement Extravasation (*n* = 429)	OR	95% CI	*p*-Value ^b^
CT vs. Combined						
Combined ^a^, *n* (%)	23 (2.3%)	12 (2.1%)	11 (2.6%)			
CT	521 (52.0%)	315 (55.0%)	206 (48.0%)	1.4	0.60–3.25	0.86
CT vs. Fluoroscopy						
Fluoroscopy ^a^, *n* (%)	457 (45.6%)	246 (42.9%)	211 (49.2%)			
CT	521 (52.0%)	315 (55.0%)	206 (48.0%)	1.32	1.02–1.70	0.15
Combined vs. Fluoroscopy						
Fluoroscopy ^a^, *n* (%)	457 (45.6%)	246 (42.9%)	211 (49.2%)			
Combined	23 (2.3%)	12 (2.1%)	11 (2.6%)	0.94	0.40–2.18	0.99

^a^: reference level. ^b^: *p*-value was adjusted for multiple comparisons.

**Table 5:** Comparison of cement extravasation location among procedure groups.

Procedure ^a^	Extravasation Location ^b^	*p*-Value
Kyphoplasty vs. BT-RFA	Intervertebral disc space vs. Posterior	0.30
Vertebroplasty vs. BT-RFA	Intervertebral disc space vs. Posterior	0.81
Kyphoplasty vs. BT-RFA	Paravertebral vs. Posterior	0.83
Vertebroplasty vs. BT-RFA	Paravertebral vs. Posterior	0.65
Kyphoplasty vs. BT-RFA	Multidirectional vs. Posterior	0.53
Vertebroplasty vs. BT-RFA	Multidirectional vs. Posterior	0.74

Abbreviations: BT-RFA, bone tumor radiofrequency ablation. ^a^: BT-RFA was utilized as the reference for the covariate (procedure type). ^b^: Posterior (spinal canal or neuroforamen) was used as reference for the outcome (extravasation location).

**Table 6:** Pre- and post-procedure pain scores (NRS) for overall cohort and each procedure group.

Procedure	Pre-Procedure NRS, Mean (SD)	Post-Procedure NRS, Mean (SD)	*p*-Value
Overall	3.15 (3.08)	3.03 (3.22)	0.39
BT-RFA	3.24 (3.15)	3.20 (3.15)	0.99
Kyphoplasty	2.89 (2.91)	3.07 (3.26)	0.34
Vertebroplasty	3.40 (3.16)	2.75 (3.25)	**0.005**

Abbreviations: NRS, numeric rating scale. Bolded *p*-values indicate statistical significance.

## Data Availability

The authors confirm that the data supporting the findings of the study are available within the article.
